# Supplementary material for: Re-definition of claudin-low as a breast cancer phenotype
Source: Nat Commun. 2020 Apr 14;11:1787. doi: 10.1038/s41467-020-15574-5 (PMC7156396; doi:10.1038/s41467-020-15574-5)
Supplement: Supplementary file 1 — Supplementary Information [file 41467_2020_15574_MOESM1_ESM.pdf]

Supplementary information

Re-definition of *claudin-low* as a breast cancer phenotype

Fougner *et al.*

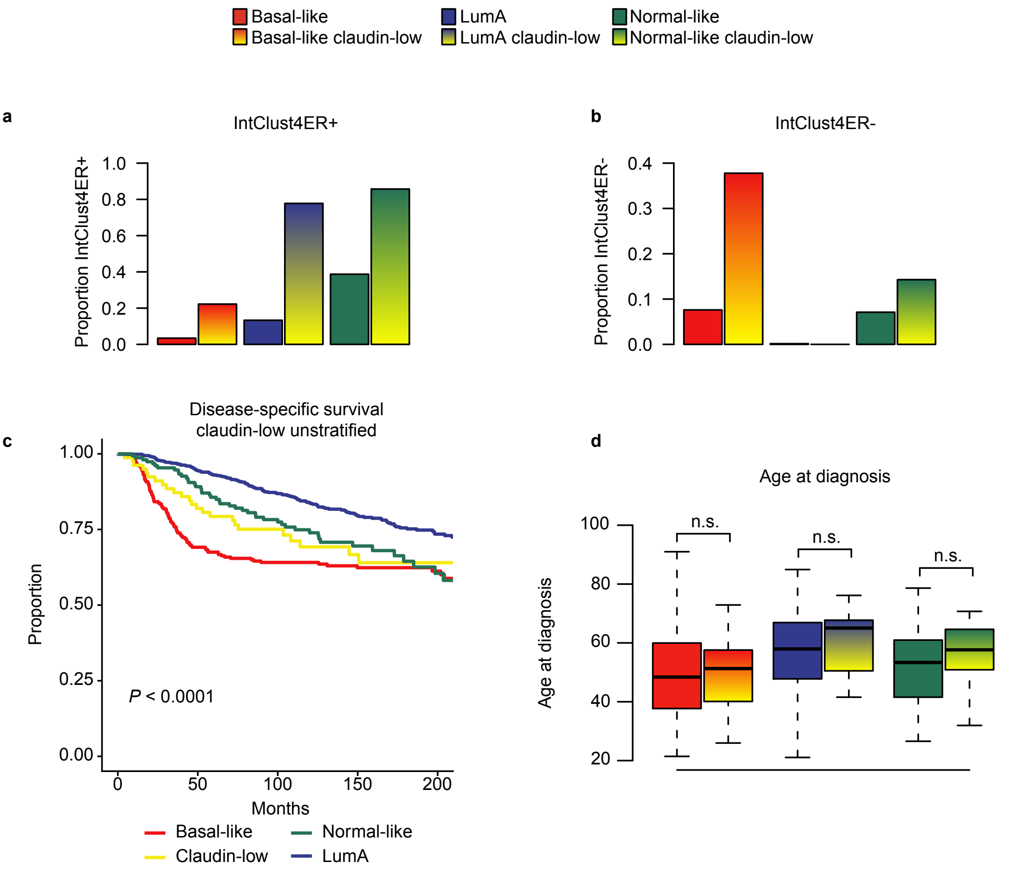

Supplementary Figure 1: Claudin-low tumors are delineated by intrinsic subtype, continued. **a** - **b** Proportion of tumors in IntClust4ER+ (a) and IntClust4ER- (b) by intrinsic subtype and claudin-low status, in the METABRIC cohort. Basal-like claudin-low tumors tended to be IntClust4ER-, whereas normal-like claudin-low and LumA claudin-low tumors tended to be IntClust4ER+. **c** Disease-specific survival in the METABRIC cohort. Patients with claudin-low tumors are here treated as a single group. The disease-specific survival of patients with claudin-low tumors was superior to that of patients with basal-like tumors, but inferior to that of patients with normal-like and LumA tumors. Disease-specific deaths and sample sizes: Basal-like n = 98 of 263, claudin-low n = 30 of 87, LumA n = 144 of 684, normal-like n = 50 of 155. Difference between groups: $P < 0.0001$, two-tailed log-rank test. **d** Age at diagnosis in claudin-low and non-claudin-low tumors. Claudin-low tumors stratified by intrinsic subtype were diagnosed at significantly different ages ($P = 0.01$, Kruskal-Wallis test), with basal-like claudin-low tumors being diagnosed at a significantly lower age than LumA claudin-low and normal-like claudin-low tumors ($P = 0.01$ and $P = 0.03$ respectively, two-tailed Wilcoxon rank-sum test). Claudin-low and non-claudin-low tumors of the same intrinsic subtype showed similar age at diagnosis (basal-like $P = 0.67$, LumA $P = 0.53$, normal-like $P = 0.052$, two-tailed Wilcoxon-rank-sum test). n.s. $P > 0.05$. Boxplot elements: center line = median, box limits = upper and lower quartiles, whiskers = 1.5 × interquartile range. **All** Sample sizes provided in Table 1. Source data are provided as a Source Data file.

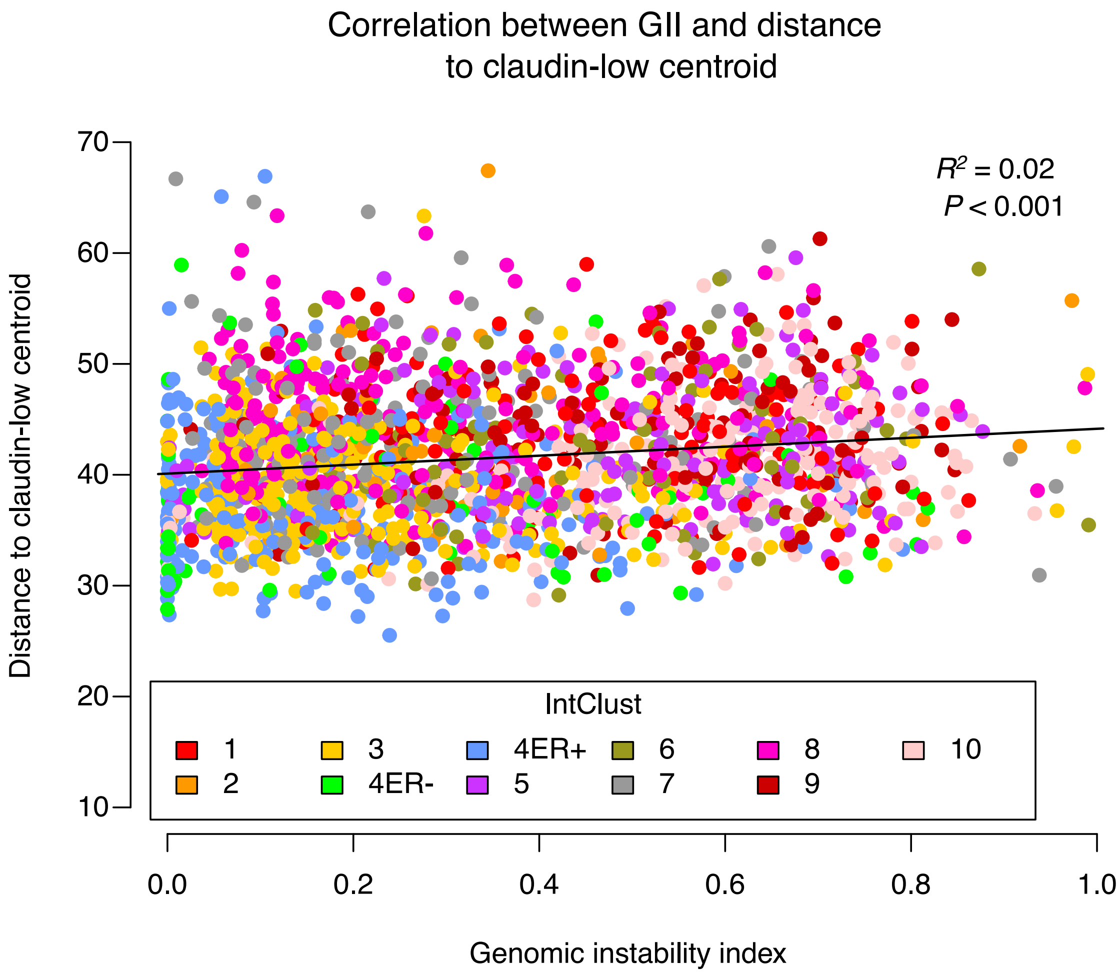

Supplementary Figure 2: Genomic instability index alone does not accurately predict correlation to the claudin-low centroid. Correlation between genomic instability index (GII) and distance to the claudin-low centroid from the nine-cell line claudin-low predictor in the METABRIC cohort. While the correlation between GII and distance to the claudin-low centroid was statistically significant, GII only accounted for 2% of the variance ($P < 0.001$, linear regression). Tumors from all intrinsic subtypes, including HER2-enriched and LumB, are included in the figure. n = 1886 biologically independent samples. Source data are provided as a Source Data file.

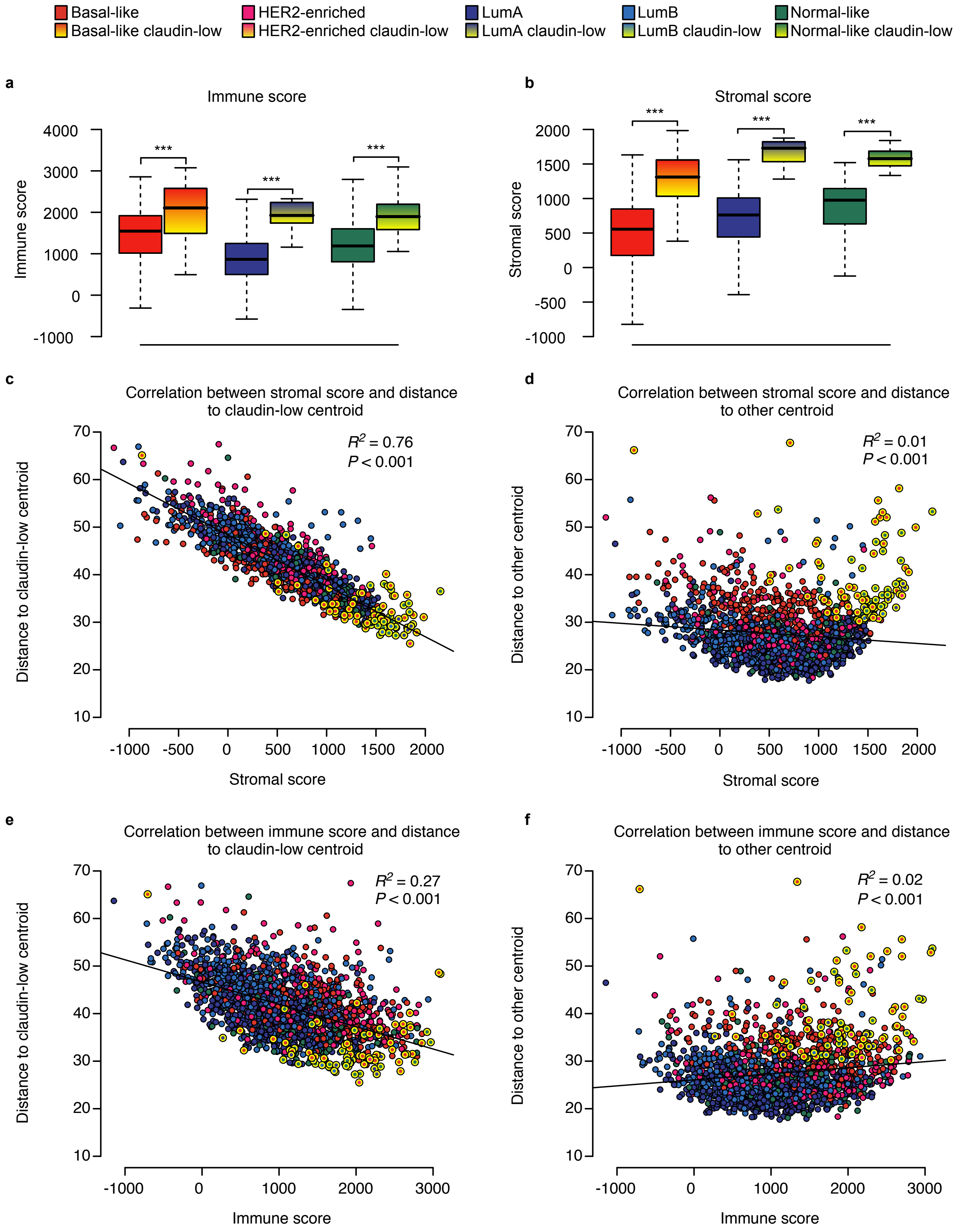

Basal-like
Basal-like claudin-low
HER2-enriched
HER2-enriched claudin-low
LumA
LumA claudin-low
LumB
LumB claudin-low
Normal-like
Normal-like claudin-low
a
Immune score
b
Stromal score
c
Correlation between stromal score and distance to claudin-low centroid
$R^2 = 0.76$
$P < 0.001$
Distance to claudin-low centroid
Stromal score
d
Correlation between stromal score and distance to other centroid
$R^2 = 0.01$
$P < 0.001$
Distance to other centroid
e
Correlation between immune score and distance to claudin-low centroid
$R^2 = 0.27$
$P < 0.001$
Immune score
f
Correlation between immune score and distance to other centroid
$R^2 = 0.02$
$P < 0.001$

Supplementary Figure 3: Claudin-low tumors show high levels of immune and stromal infiltration. a - b Immune and stromal score, from ESTIMATE, in claudin-low and non-claudin-low tumors in the METABRIC cohort. Claudin-low tumors of all subtypes had higher levels of immune and stromal infiltration than non-claudin-low tumors of the same subtype ($P < 0.001$ for all, two-tailed Wilcoxon rank-sum test). **c - d** Relationship between stromal score and Euclidean distance to the claudin-low centroid (**c**) and the other centroid (**d**) from the nine-cell line predictor (linear regression). An inverse correlation between stromal score and distance to the claudin-low centroid was observed ($R^2 = 0.76$). **e - f** Relationship between immune score and Euclidean distance to the claudin-low centroid (**e**) and the other centroid (**f**) from the nine-cell line predictor (linear regression). An inverse correlation between immune score and distance to the claudin-low centroid was observed ($R^2 = 0.27$). **c – f** $P < 0.001$ for all linear regressions. **All** *** $P < 0.001$. Boxplot elements: center line = median, box limits = upper and lower quartiles, whiskers = 1.5 × interquartile range. Sample sizes provided in Table 1 (HER2-enriched and LumB tumors are included in panels **c** - **f**). Source data are provided as a Source Data file.

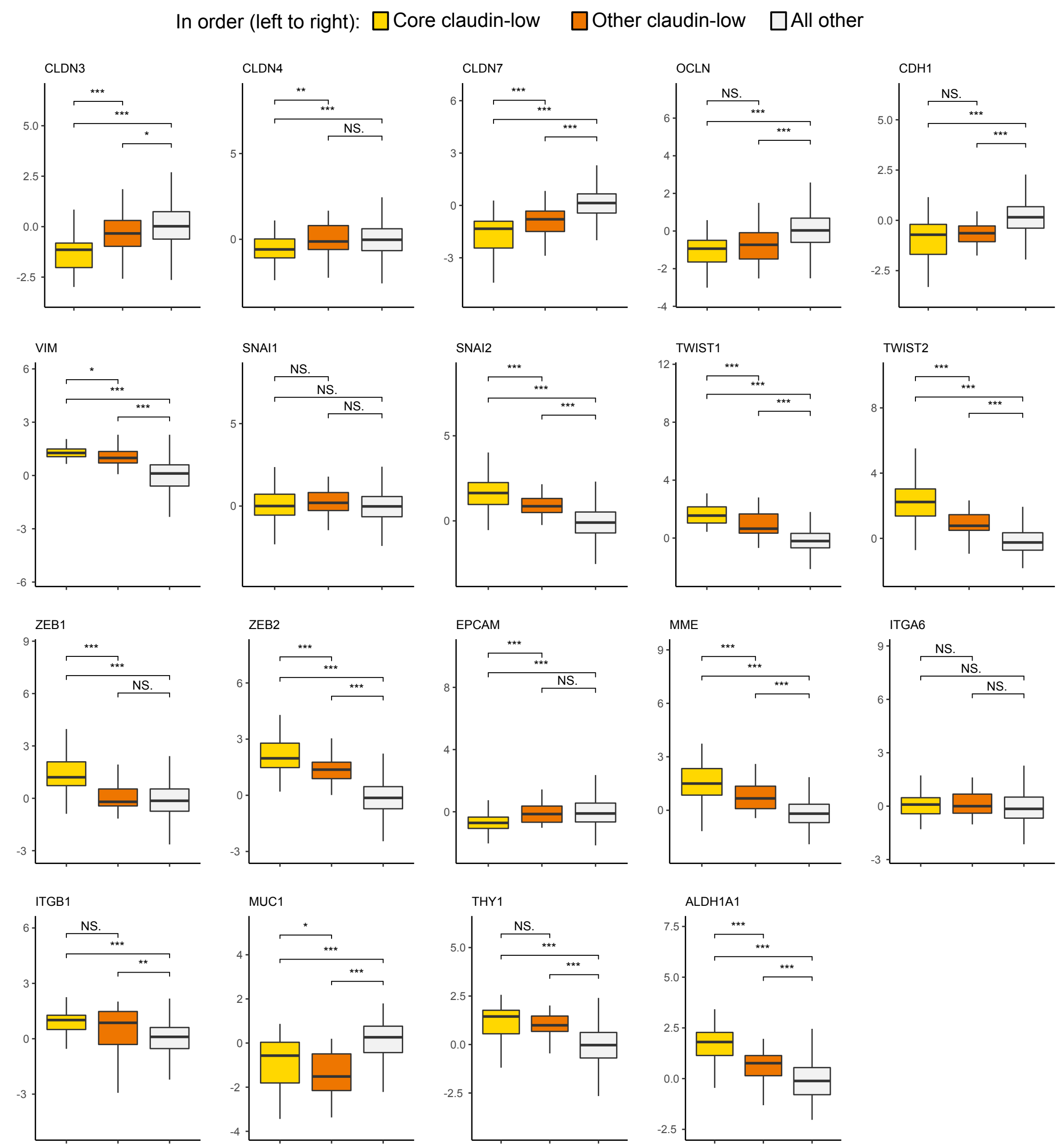

Supplementary Figure 4: CoreCL tumors show gene expression features consistent with the claudin-low phenotype. Gene expression (*log2*) for all 19 genes in the condensed claudin-low gene list, in the METABRIC cohort (n = 1886 biologically independent samples), separated into CoreCL, OtherCL and all other tumors. CoreCL tumors showed gene expression characteristics in line with those previously described for claudin-low tumors. OtherCL tumors showed some claudin-low characteristics, albeit to a lesser degree than CoreCL tumors. Two-tailed Wilcoxon rank-sum test used for significance testing. NS. $P > 0.05$, * $P < 0.05$, ** $P < 0.01$, *** $P < 0.001$. Boxplot elements: center line = median, box limits = upper and lower quartiles, whiskers = 1.5 × interquartile range. CoreCL n = 79, OtherCL n = 30, non-claudin-low n = 1777 biologically independent samples. Source data are provided as a Source Data file.

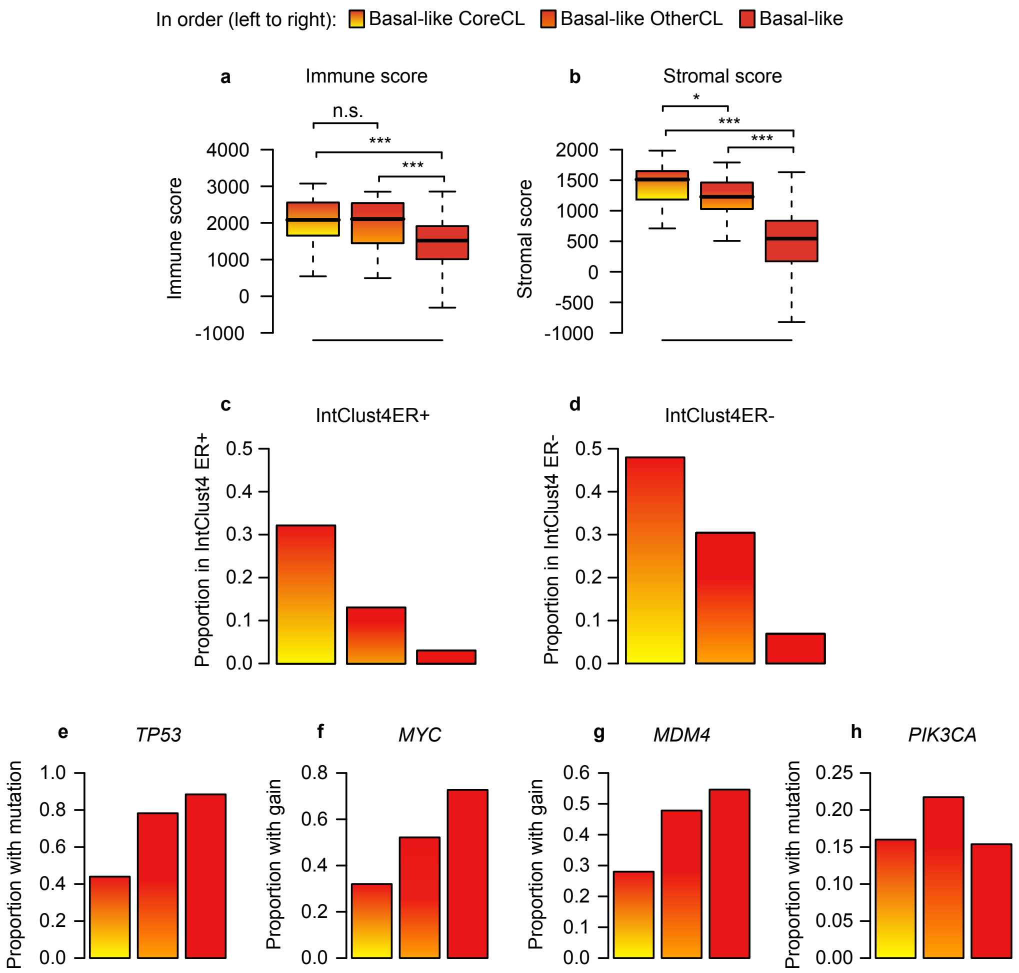

Supplementary Figure 5: Basal-like OtherCL tumors may be inappropriately classified as claudin-low, continued. a - b Immune and stromal score in basal-like core claudin-low tumors, basal-like other claudin-low tumors, and basal-like non-claudin-low tumors in the METABRIC cohort. Basal-like CoreCL and OtherCL tumors showed higher immune and stromal infiltration than basal-like non-claudin-low tumors ($P < 0.001$ for all, two-tailed Wilcoxon rank-sum test). **c - d** Proportion of basal-like tumors in IntClust4ER+ (**c**) and IntClust4ER- (**d**) by claudin-low status. The majority of basal-like CoreCL tumors were classified as IntClust4, with an overweight of tumors not expressing ER. **e - h** Proportion of tumors with mutation or copy number gain in key genes. Basal-like CoreCL tumors showed lower rates of *TP53* mutation (**e**), *MYC* gain (**f**) and *MDM4* gain (**g**) than basal-like OtherCL and non-claudin-low basal-like tumors. This trend was however not evident in the distribution of *PIK3CA* mutations (**h**). **All** n.s. $P > 0.05$, * $P < 0.05$, *** $P < 0.001$. Boxplot elements: center line = median, box limits = upper and lower quartiles, whiskers = 1.5 × interquartile range. Basal-like CoreCL $n = 25$, basal-like OtherCL $n = 23$, basal-like non-claudin-low $n = 260$ biologically independent samples. Source Data are provided as a Source Data file.

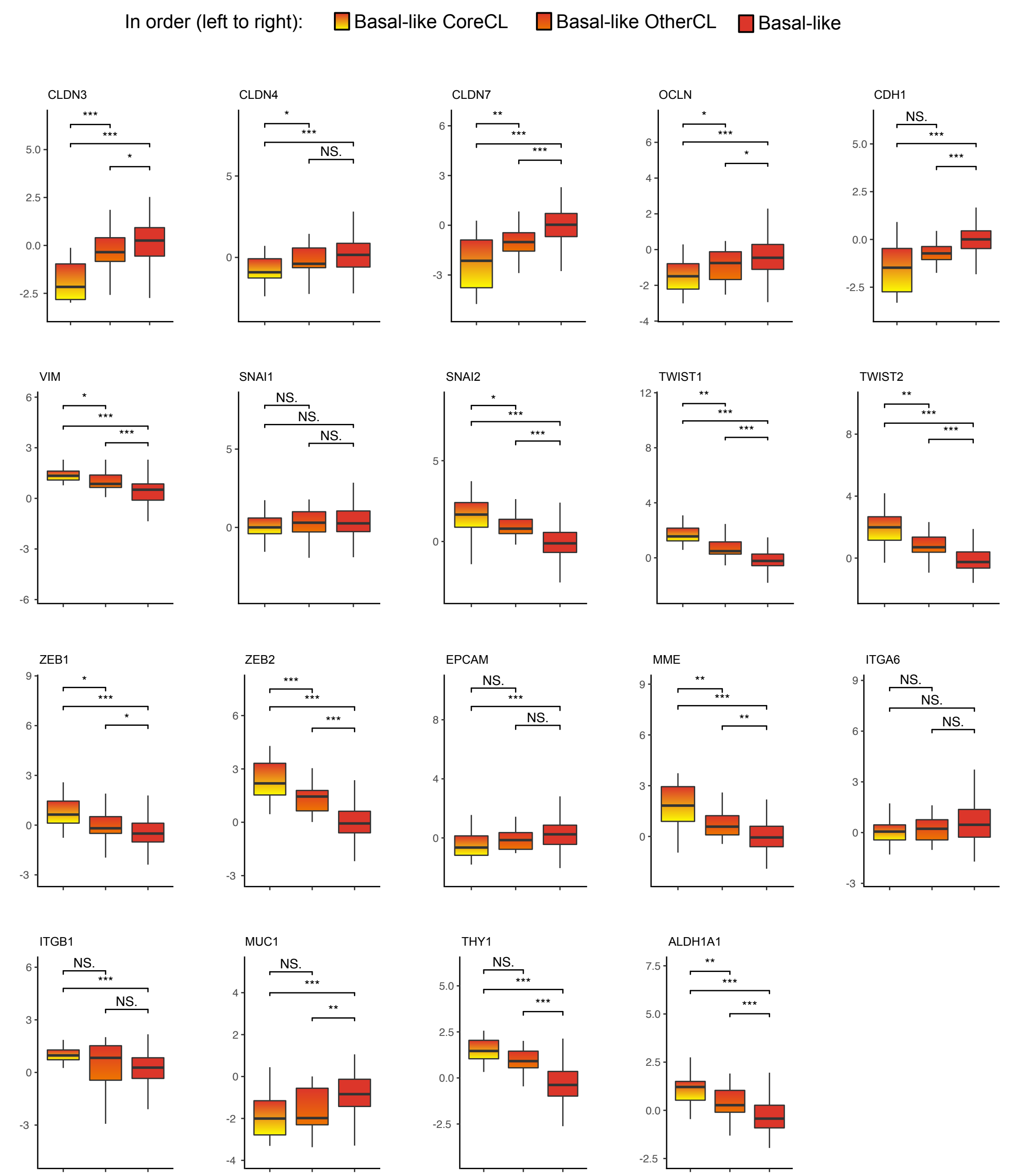

Supplementary Figure 6: Basal-like CoreCL tumors show claudin-low gene expression characteristics. Gene expression (*log2*) for all 19 genes in the condensed claudin-low gene list for basal-like core claudin-low tumors, basal-like other claudin-low tumors and non-claudin-low basal-like tumors in the METABRIC cohort. Two-tailed Wilcoxon rank-sum test used for significance testing. NS. $P > 0.05$, * $P < 0.05$, ** $P < 0.01$, *** $P < 0.001$. Boxplot elements: center line = median, box limits = upper and lower quartiles, whiskers = 1.5 × interquartile range. Basal-like CoreCL n = *25*, basal-like OtherCL n = 23, basal-like non-claudin-low n = 260 biologically independent samples. Source data are provided as a Source Data file.

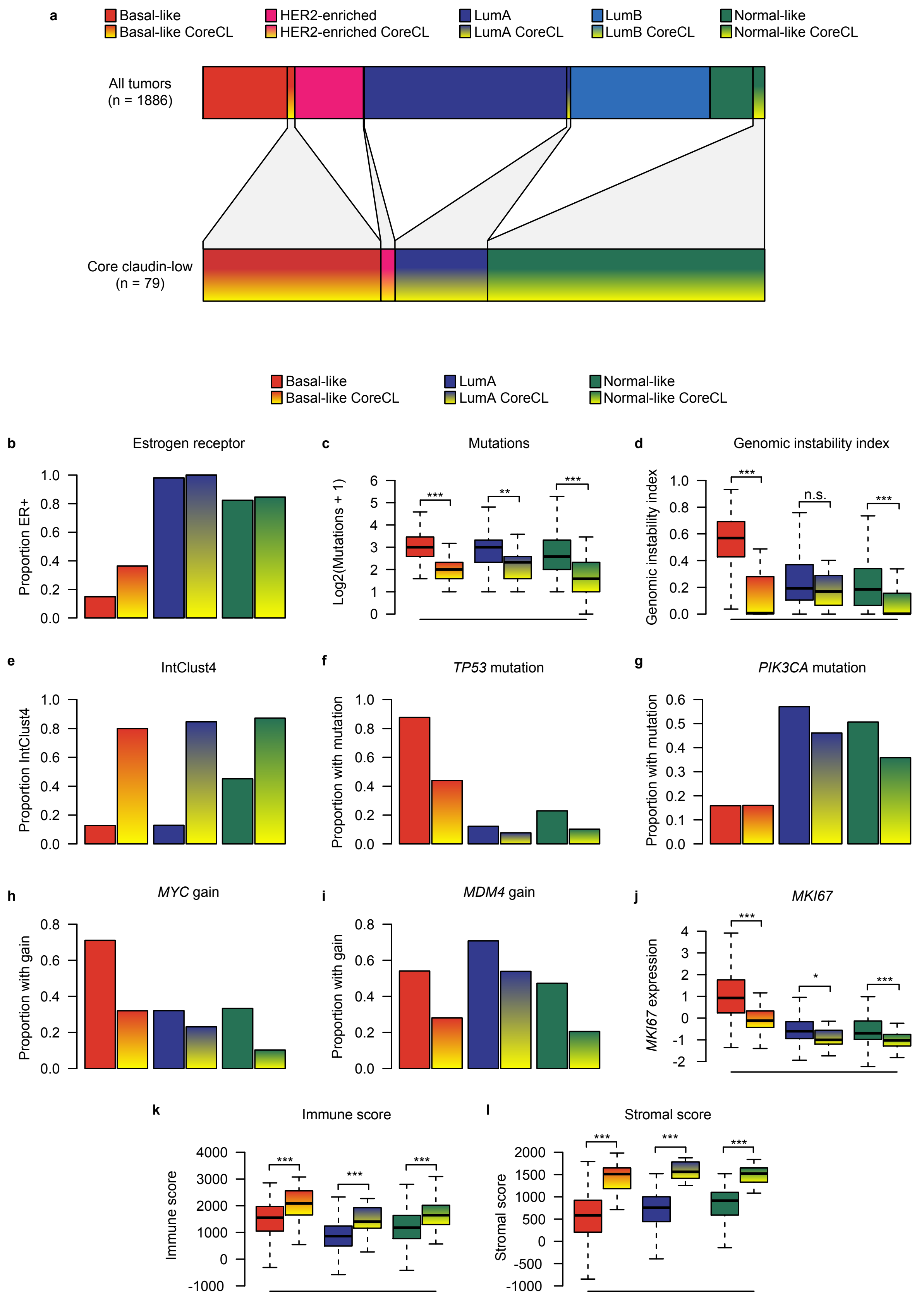
a
Basal-like
Basal-like CoreCL
HER2-enriched
HER2-enriched CoreCL
LumA
LumA CoreCL
LumB
LumB CoreCL
Normal-like
Normal-like CoreCL
All tumors (n = 1886)
Core claudin-low (n = 79)
Basal-like
Basal-like CoreCL
LumA
LumA CoreCL
Normal-like
Normal-like CoreCL
b
Estrogen receptor
Proportion ER+
c
Mutations
Log2(Mutations + 1)
***
**
d
Genomic instability index
n.s.
e
IntClust4
Proportion IntClust4
f
TP53 mutation
Proportion with mutation
g
PIK3CA mutation
h
MYC gain
Proportion with gain
i
MDM4 gain
j
MKI67
MKI67 expression
*
k
Immune score
l
Stromal score

Supplementary Figure 7: CoreCL tumors are more homogeneous than claudin-low tumors identified by the nine-cell line predictor. **a** Distribution of intrinsic subtypes in the METABRIC cohort for all tumors (top bar, *n* = 1886) and for CoreCL tumors only (bottom bar, *n* = 79). **b** Distribution of estrogen receptor-positivity. **c** Number of mutations in the panel of 173 sequenced genes. CoreCL tumors showed lower mutational rates than non-claudin-low tumors of the same subtype (basal-like $P < 0.001$, LumA $P = 0.009$, normal-like $P < 0.001$, two-tailed Wilcoxon rank-sum test). **d** Distribution of genomic instability index (GII). Basal-like and normal-like CoreCL tumors showed lower levels of genomic instability than non-claudin-low tumors of the same subtype (basal-like $P < 0.001$, LumA $P = 0.17$, normal-like $P < 0.001$, two-tailed Wilcoxon rank-sum test). **e** Distribution of allocation to the IntClust4 subtype. **f** Distribution of *TP53* mutations. **g** Distribution of *PIK3CA* mutations. **h** Distribution of *MYC* gain. **i** *MDM4* gain. **j** Distribution of *MKI67* gene expression (*log2*). CoreCL tumors consistently expressed lower levels of *MKI67* compared to non-claudin-low counterparts (basal-like $P < 0.001$, LumA $P = 0.01$, normal-like $P < 0.001$, two-tailed Wilcoxon rank-sum test). **k - l** Distribution of immune and stromal score from ESTIMATE. CoreCL tumors had higher immune and stromal score than non-claudin-low tumors ($P < 0.001$ for all, two-tailed Wilcoxon-rank sum test). **All** There was a reduced variability in the characteristics of basal-like CoreCL tumors compared to basal-like claudin-low tumors as classified by the nine-cell line predictor. The characteristics of LumA CoreCL and normal-like CoreCL tumors were similar to the characteristics of LumA claudin-low and normal-like claudin-low tumors as classified by the nine-cell line predictor. n.s. $P > 0.05$, * $P < 0.05$, ** $P < 0.01$, *** $P < 0.001$. Boxplot elements: center line = median, box limits = upper and lower quartiles, whiskers = 1.5 × interquartile range. Here, OtherCL tumors are treated as non-claudin-low. Basal-like CoreCL *n = 25,* basal-like non-claudin-low *n* = 283, HER2-enriched CoreCL *n* = 2, HER2-enriched non-claudin-low *n* = 231, LumA CoreCL *n* = 13, LumA non-claudin-low *n* = 680, LumB CoreCL *n* = 0, LumB non-claudin-low *n* = 469, normal-like CoreCL *n* = 39, normal-like non-claudin-low *n* = 144 biologically independent samples. Source data are provided as a Source Data file.

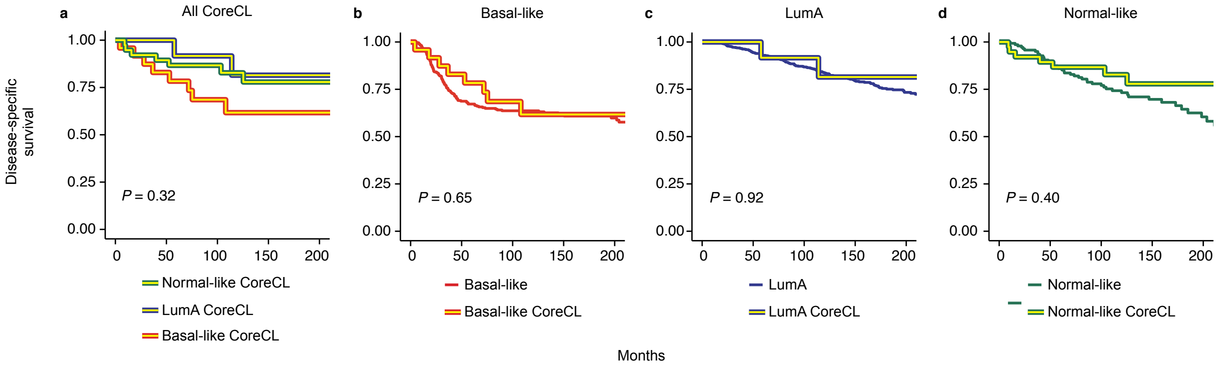

Supplementary Figure 8: No evidence of CoreCL-status as an indicator of poor prognosis in the METABRIC cohort. a Disease-specific survival in basal-like CoreCL, LumA CoreCL, and normal-like CoreCL tumors in the METABRIC cohort. Survival trends recapitulated the patterns seen in non-claudin-low tumors, although differences in disease-specific survival between CoreCL tumors stratified by intrinsic subtype did not approach statistical significance (*P* = 0.32, two-tailed log-rank test). **b - d** Disease specific survival in CoreCL and non-claudin-low basal-like (**b**), LumA (**c**) and normal-like (**d**) tumors. Significant differences between CoreCL and non-claudin-low tumors were not found (basal-like *P* = 0.65, LumA *P* = 0.92, normal-like *P* = 0.40, two-tailed log-rank test). **All** Here, OtherCL tumors are treated as non-claudin-low. Basal-like CoreCL *n = 25,* basal-like non-claudin-low *n* = 283, LumA CoreCL *n* = 13, LumA non-claudin-low *n* = 680, normal-like CoreCL *n* = 39, normal-like non-claudin-low *n* = 144 biologically independent samples. Disease-specific deaths: Basal-like CoreCL *n* = 8 of 25, basal-like non-claudin-low *n* = 109 of 283, LumA CoreCL *n* = 3 of 13, LumA non-claudin-low *n* = 144 of 680, normal-like CoreCL *n* = 9 of 39, normal-like non-claudin-low *n* = 46 of 144. Source data are provided as a Source Data file.

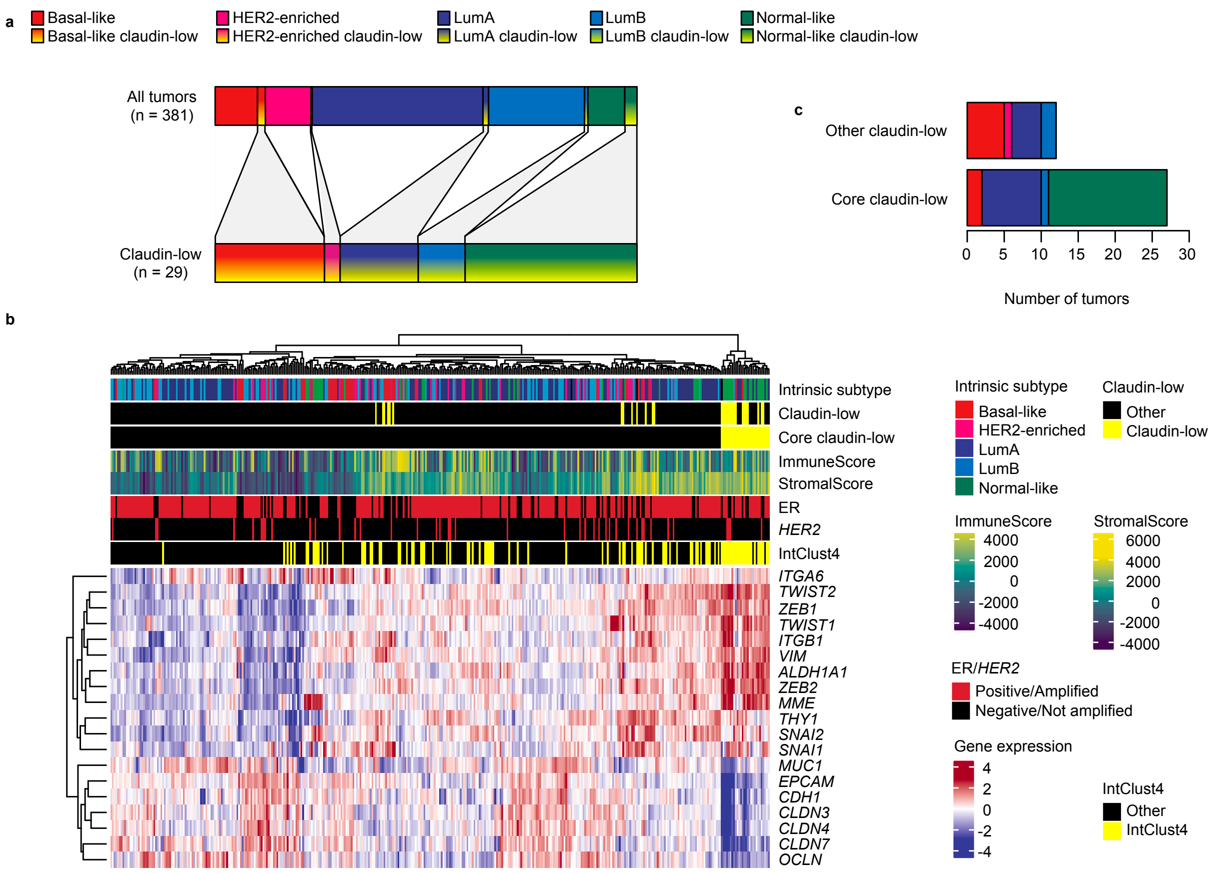

Supplementary Figure 9: Claudin-low tumors in the Oslo2 cohort recapitulate characteristics observed in the METABRIC cohort. a Distribution of intrinsic subtypes in the Oslo2 cohort for all tumors (top bar, *n* = 381 biologically independent samples) and for claudin-low tumors, as defined by the nine-cell line predictor (bottom bar, *n* = 29 biologically independent samples). Most claudin-low tumors were either basal-like, LumA, or normal-like. **b** Heatmap of gene expression values (*log2*) for the condensed claudin-low gene list in the Oslo2 cohort (*n* = 381 biologically independent samples). Hierarchical clustering identified a core claudin-low cluster ($P < 0.001$, SigClust) with similar characteristics to those observed in the METABRIC cohort. Copy number data was not available, however, the representation of IntClust4 in the core claudin-low cluster implies genomic stability in the group. **c** Distribution of subtypes in core and other claudin-low tumors in the Oslo2 cohort. The distribution of subtypes was similar to that seen in the METABRIC cohort, with a slightly larger variation in the intrinsic subtypes of OtherCL tumors. Basal-like CoreCL *n* = 2, basal-like OtherCL *n* = 5, HER2-enriched OtherCL *n* = 1, LumA CoreCL *n* = 8, LumA OtherCL *n* = 4, LumB CoreCL *n* = 1, LumB OtherCL *n* = 2, normal-like CoreCL *n* = 16 biologically independent samples. *n* = 1 CoreCL and *n* = 1 OtherCL sample without available intrinsic subtype. **All** Source data are provided as a Source Data file.

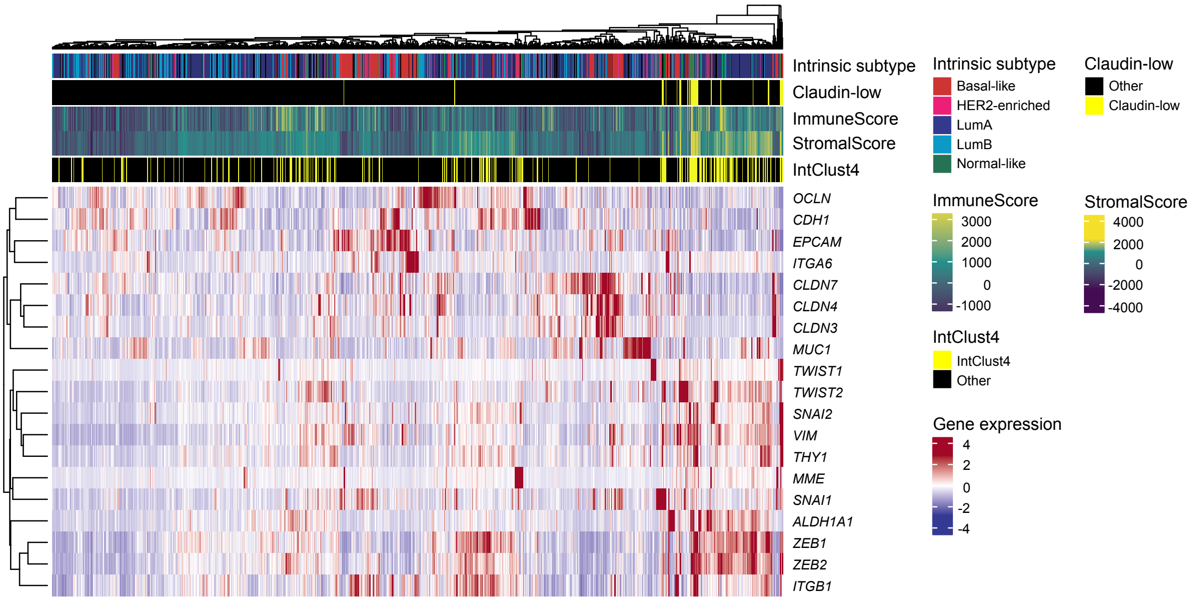

Supplementary Figure 10: No core claudin-low cluster in the TCGA-BRCA cohort. Heatmap of gene expression values (*log2*) for the condensed claudin-low gene list in the TCGA-BRCA cohort (n = 1082 biologically independent samples). No core claudin-low cluster emerged. Source data are provided as a Source Data file.

Gene	EntrezID	Characteristic	Expected regulation in claudin-low
CLDN3	1365	Cell-cell adhesion & tight junction	Down
CLDN4	1364	Cell-cell adhesion & tight junction	Down
CLDN7	1366	Cell-cell adhesion & tight junction	Down
OCLN	100506658	Cell-cell adhesion & tight junction	Down
CDH1	999	Cell-cell adhesion & tight junction	Down
VIM	7431	EMT	Up
SNAI1	6615	EMT	Up
SNAI2	6591	EMT	Up
TWIST1	7291	EMT	Up
TWIST2	117581	EMT	Up
ZEB1	6935	EMT	Up
ZEB2	9839	EMT	Up
EPCAM	4072	Stem cell & epithelial differentiation	Down
MUC1	4582	Stem cell & epithelial differentiation	Down
MME	4311	Stem cell & epithelial differentiation	Up
ITGA6	3655	Stem cell & epithelial differentiation	Up
ITGB1	3688	Stem cell & epithelial differentiation	Up
THY1	7070	Stem cell & epithelial differentiation	Up
ALDH1A1	216	Stem cell & epithelial differentiation	Up

Supplementary Table 1: A condensed claudin-low gene list. A list of 19 genes pathognomonic to the claudin-low phenotype. Characteristics/functions listed in the "Characteristic" column are guiding; listed genes may be representative of multiple functions.
