# Supplementary material for: Re-definition of claudin-low as a breast cancer phenotype
Source: Nat Commun. 2020 Apr 14;11:1787. doi: 10.1038/s41467-020-15574-5 (PMC7156396; doi:10.1038/s41467-020-15574-5)
Supplement: Supplementary file 3 — Description of Additional Supplementary Files [file 41467_2020_15574_MOESM3_ESM.pdf]

Description of Additional Supplementary Files

File Name: Supplementary Data 1
Description: **METABRIC cohort sample information.** Table containing relevant annotations for samples and patients in the METABRIC cohort.

File Name: Supplementary Data 2
Description: **Comparative rates of mutations and CNAs in claudinlow and non-claudin-low tumors in the METABRIC cohort.** No mutations were found at a significantly higher rate in claudin-low tumors, stratified by intrinsic subtype, than in non-claudin-low tumors of the same subtype (two-tailed Fisher's exact test, Bonferroni corrected). In analyses of core claudin-low tumors (see subheading "A condensed gene list refines claudin-low classification"), OtherCL tumors are treated as non-claudin-low.

File Name: Supplementary Data 3
Description: **Oslo2 cohort sample information.** Table containing relevant annotations for samples and patients in the Oslo2 cohort.

File Name: Supplementary Data 4
Description: **TCGA-BRCA cohort sample information.** Table containing relevant annotations for samples and patients in the TCGA-BRCA cohort.
