# Supplementary material for: Re-definition of claudin-low as a breast cancer phenotype
Source: Nat Commun. 2020 Apr 14;11:1787. doi: 10.1038/s41467-020-15574-5 (PMC7156396; doi:10.1038/s41467-020-15574-5)
Supplement: Supplementary file 8 — Reporting Summary [file 41467_2020_15574_MOESM8_ESM.pdf]

nature research

Corresponding author(s): Therese Sørlie

Last updated by author(s): Feb 20, 2020

Reporting Summary

Nature Research wishes to improve the reproducibility of the work that we publish. This form provides structure for consistency and transparency in reporting. For further information on Nature Research policies, see Authors & Referees and the Editorial Policy Checklist .

Statistics

For all statistical analyses, confirm that the following items are present in the figure legend, table legend, main text, or Methods section.

n/a	Confirmed	
☐	☒	The exact sample size (n) for each experimental group/condition, given as a discrete number and unit of measurement
☐	☒	A statement on whether measurements were taken from distinct samples or whether the same sample was measured repeatedly
☐	☒	The statistical test(s) used AND whether they are one- or two-sided<br>*Only common tests should be described solely by name; describe more complex techniques in the Methods section.*
☐	☒	A description of all covariates tested
☐	☒	A description of any assumptions or corrections, such as tests of normality and adjustment for multiple comparisons
☐	☒	A full description of the statistical parameters including central tendency (e.g. means) or other basic estimates (e.g. regression coefficient) AND variation (e.g. standard deviation) or associated estimates of uncertainty (e.g. confidence intervals)
☐	☒	For null hypothesis testing, the test statistic (e.g. F, t, r) with confidence intervals, effect sizes, degrees of freedom and P value noted<br>*Give P values as exact values whenever suitable.*
☒	☐	For Bayesian analysis, information on the choice of priors and Markov chain Monte Carlo settings
☐	☒	For hierarchical and complex designs, identification of the appropriate level for tests and full reporting of outcomes
☐	☒	Estimates of effect sizes (e.g. Cohen's d, Pearson's r), indicating how they were calculated

Our web collection on statistics for biologists contains articles on many of the points above.

Software and code

Policy information about availability of computer code

Data collection	No custom code was used to collect data in this study.
Data analysis	All analyses performed in this study are described at www.github.com/clfougner/ClaudinLow<br><br>All analyses were run in R Version 3.6.0. The R packages used in the analyses are:<br>- Genefu v2.16.0<br>- ESTIMATE v1.0.13<br>- ComplexHeatmap v2.0.0<br>- SigClust v1.1.0<br>- Biobase v2.44.0<br>- GEOquery v2.52.0<br>- Survival v2.44-1.1<br>- Survminer v0.4.5<br>- ggsci v2.9<br>- circlize v0.4.7<br>- ggplot2 v3.2.1<br>- ggsignif v0.6.0<br>- gtools v3.8.1<br>- gridExtra v2.3

For manuscripts utilizing custom algorithms or software that are central to the research but not yet described in published literature, software must be made available to editors/reviewers. We strongly encourage code deposition in a community repository (e.g. GitHub). See the Nature Research guidelines for submitting code & software for further information.

Data

Policy information about availability of data

All manuscripts must include a data availability statement. This statement should provide the following information, where applicable:

- Accession codes, unique identifiers, or web links for publicly available datasets
- A list of figures that have associated raw data
- A description of any restrictions on data availability

The data used in this study are available through cBioportal (METABRIC [1], TCGA [2]), GSE80999 [3], supplementary tables 2 and 3 in Curtis et al. [4] and the repository [5] associated with Pereira et al. [6] Histological classification of the METABRIC cohort may be available upon request to Mukherjee et al. [7] Detailed instructions for gathering data can be found in the repository [8] associated with this study. The source data underlying each figure are provided as a Source Data file.

[1] https://www.cbioportal.org/study/summary?id=brca_metabric
[2] https://www.cbioportal.org/study/summary?id=brca_tcga_pan_can_atlas_2018]
[3] https://www.ncbi.nlm.nih.gov/geo/query/acc.cgi?acc=GSE80999
[4] Curtis, C. et al. The genomic and transcriptomic architecture of 2,000 breast tumours reveals novel subgroups. Nature 486, 346 (2012).
[5] https://github.com/cclab-brca/mutationalProfiles/tree/master/Data
[6] Pereira, B. et al. The somatic mutation profiles of 2,433 breast cancers refine their genomic and transcriptomic landscapes. Nat. Commun. 7, 11479 (2016).
[7] Mukherjee, A. et al. Associations between genomic stratification of breast cancer and centrally reviewed tumour pathology in the METABRIC cohort. NPJ breast cancer 4, 5 (2018).
[8] https://github.com/clfougner/ClaudinLow

Field-specific reporting

Please select the one below that is the best fit for your research. If you are not sure, read the appropriate sections before making your selection.

- [x] Life sciences
- [ ] Behavioural & social sciences
- [ ] Ecological, evolutionary & environmental sciences

For a reference copy of the document with all sections, see nature.com/documents/nr-reporting-summary-flat.pdf

Life sciences study design

All studies must disclose on these points even when the disclosure is negative.

Sample size	All samples with required data publicly available from the METABRIC, Oslo2, and TCGA-BRCA cohorts were included.
Data exclusions	HER2-enriched and Luminal B tumors from the METABRIC cohort were not studied in depth as there were only 2 and 3 claudin-low tumors from each group, respectively (not pre-established).
Replication	N/A as no experiments were performed in this study (only deterministic analyses of publicly available datasets).
Randomization	N/A as this study contained no experimental intervention, and there was consequently no allocation to experimental groups.
Blinding	N/A as there were no experimental interventions to which investigators could be blinded.

Reporting for specific materials, systems and methods

We require information from authors about some types of materials, experimental systems and methods used in many studies. Here, indicate whether each material, system or method listed is relevant to your study. If you are not sure if a list item applies to your research, read the appropriate section before selecting a response.

Materials & experimental systems

n/a	Involved in the study	
[x]	[ ]	Antibodies
[x]	[ ]	Eukaryotic cell lines
[x]	[ ]	Palaeontology
[x]	[ ]	Animals and other organisms
[ ]	[x]	Human research participants
[x]	[ ]	Clinical data

Methods

n/a	Involved in the study	
[x]	[ ]	ChIP-seq
[x]	[ ]	Flow cytometry
[x]	[ ]	MRI-based neuroimaging

Human research participants

Policy information about studies involving human research participants

Population characteristics	Breast cancer patients from three publicly available cohorts were included. These cohorts primarily include female patients sampled from European and North American populations. The detailed characteristics of each cohort are described in their respective publications [1-4].<br><br>[1] Curtis, C. et al. The genomic and transcriptomic architecture of 2,000 breast tumours reveals novel subgroups. Nature 486, 346 (2012).<br>[2] The Cancer Genome Atlas Network. Comprehensive molecular portraits of human breast tumours. Nature 490, 61 (2012).<br>[3] Hoadley, K. A. et al. Cell-of-origin patterns dominate the molecular classification of 10,000 tumors from 33 types of cancer. Cell 173, 291–304 (2018).<br>[4] Aure, M. R. et al. Integrative clustering reveals a novel split in the luminal A subtype of breast cancer with impact on outcome. Breast Cancer Res. 19, 44 (2017).
Recruitment	No patients were recruited as part of this study.
Ethics oversight	This study is an analysis of de-identified publicly available data, and no ethical approvals were therefore required. The ethical approvals for the cohorts used in this study are available in their respective publications [1-4].<br><br>[1] Curtis, C. et al. The genomic and transcriptomic architecture of 2,000 breast tumours reveals novel subgroups. Nature 486, 346 (2012).<br>[2] The Cancer Genome Atlas Network. Comprehensive molecular portraits of human breast tumours. Nature 490, 61 (2012).<br>[3] Hoadley, K. A. et al. Cell-of-origin patterns dominate the molecular classification of 10,000 tumors from 33 types of cancer. Cell 173, 291–304 (2018).<br>[4] Aure, M. R. et al. Integrative clustering reveals a novel split in the luminal A subtype of breast cancer with impact on outcome. Breast Cancer Res. 19, 44 (2017).

Note that full information on the approval of the study protocol must also be provided in the manuscript.
